# Ampullary Cancer: Histological Subtypes, Markers, and Clinical Behaviour—State of the Art and Perspectives

**DOI:** 10.3390/curroncol30070507

**Published:** 2023-07-22

**Authors:** Gennaro Nappo, Niccola Funel, Virginia Laurenti, Elisabetta Stenner, Silvia Carrara, Silvia Bozzarelli, Paola Spaggiari, Alessandro Zerbi

**Affiliations:** 1 Pancreatic Surgery Unit, Humanitas Clinical and Research Center—IRCCS, 20089 Rozzano, Italyalessandro.zerbi@humanitas.it (A.Z.); 2 Department of Biomedical Sciences, Humanitas University, Via Rita Levi Montalcini 4, Pieve Emanuele, 20090 Milan, Italy; 3 USL Toscana Nordovest, Chemical-Clinical Analysis Laboratory, Department of Diagnostics, 56121 Pisa, Italy; niccola.funel@uslnordovest.toscana.it (N.F.); elisabetta.stenner@uslnordovest.toscana.it (E.S.); 4 Endoscopic Unit, Humanitas Clinical and Research Center—IRCCS, 20089 Rozzano, Italy; 5 Medical Oncology and Hematology Unit, Humanitas Cancer Center, Humanitas Clinical and Research Center—IRCCS, 20089 Rozzano, Italy; 6 Pathology Unit, Humanitas Clinical and Research Center—IRCCS, 20089 Rozzano, Italy; paola.spaggiari@humanitas.it

**Keywords:** ampullary adenocarcinomas (AAC), subtypes, molecular markers, immuno-histochemistry, clinical behaviours, clinical management, CK7, CK20, CDX2

## Abstract

There are different cancers in the peri-ampullary region, including pancreatic ductal adenocarcinoma (PDAC), duodenum cancers (DCs), and ampullary adenocarcinoma (AAC). Here, significant morphological–molecular characterizations should be necessary for the distinction of primary tumours and classifications of their subtypes of cancers. The sub classification of AACs might include up to five different variants, according to different points of view, concerning the prevalence of the two more-cellular components found in the ampulla. In particular, regarding the AACs, the most important subtypes are represented by the intestinal (INT) and the pancreato-biliary (PB) ones. The subtyping of AACs is essential for diagnosis, and their identifications have been impacting clinical management responses to treatments and overall survival (os) after surgery. Pb is associated with a worse clinical outcome. Otherwise, the criteria, through which are possible to attribute its subtype classification, are not well established. A triage of immune markers represented by CK7, CK20, and CDX-2 seem to represent the best compromise in order to split the cohort of AAC patients in the INT and PB groups. The test of choice for the sub-classification of AACs is represented by the immuno-histochemical approach, in which its molecular classification acquires its diagnostic, predictive, and prognostic value for both the INT and PB patients.

## 1. Introduction

Ampullary adenocarcinoma (AAC) is a rare disease, accounting for only 0.2% of gastrointestinal malignancies and 6% of periampullary tumours [1]. The ampullary region is a very complex anatomical region, representing the convergence of multiple structures. It can be divided into three different compartments, each of them with specific histological features [2]: the distal common bile duct and pancreatic duct, which are lined by pancreatobiliary-type epithelium; the Vater’s papilla, which is covered by the small intestinal epithelium; the wall of the ampulla with the Oddi musculature and ductules that are lined by the pancreatobiliary-type epithelium [3]. AAC can arise from each of these structures, developing different histological subtypes according to the epithelium of origin: pancreatobiliary (PB) when it originates from the pancreatobiliary epithelium of the ampullary region, or intestinal (INT) when it originates from the intestinal epithelium [4]. The objective of this article is to critically review the available literature concerning the two histological subtypes of AAC, particularly focusing on (a) their histological and immunohistochemical features; (b) their impacts on prognoses; (c) the roles of adjuvant treatments for each subtype. 

## 2. Definition of Different Subtypes of AAC

Firstly, in 1913, Outerbridge [5] pointed out that ampullary adenocarcinoma could theoretically arise from different parts of the papilla: the duodenal mucosa, the epithelium of the common pancreaticobiliary channel, the epithelium of the lower bile duct, or the epithelium of the lower pancreatic duct. A great step forward for the understanding of ampullary adenocarcinoma was the publication by kimura et al. in 1994 [4], describing two distinct histological subtypes for the first time: (a) the intestinal subtype that originated from the intestinal epithelium of the papilla and (b) the pancreatobiliary subtype that originated from the epithelium of the wirsung duct. Since this publication, several studies have adopted this distinction [6,7,8,9,10]. Currently, the last classifications of ampullary cancer were distinguished separately into the intestinal (int) and pancreato-biliary (pb) subtypes [11].

These two kinds of ampullary cancer have evidently different histological and biological features. The intestinal AAC subtype is characterized by glandular units that are lined by variably differentiated intestinal-type epithelium with morphological features similar to conventional colonic adenocarcinomas [12]. From a pathogenic point of view, the development of INT seems to be related to a classical evolution through an adenoma–dysplasia–adenocarcinoma sequence, commonly observed in the pathogenesis of colon–rectal cancer [10,13,14,15,16]. Conversely, the PB variant is characterized by variably differentiated glands: clusters or singly dispersed non-stratified cuboidal or low columnar eosinophilic epithelium containing round to oval, irregular, hypochromatic, or hyperchromatic nuclei with abundant desmoplastic stroma, similarly to pancreato-biliary carcinomas [17]. Differently from INT, they rarely have an associated adenomatous component, but they evolve from precursor INTRADUCTAL IN SITU CARCINOMA in an analogous dysplasia–adenocarcinoma sequence [10,13,18]. Regarding the two subtypes, PB has a higher incidence (PB 70% vs. INT 30%)); moreover, int is generally largest (mean size 4.5 cm), compared with pb (2.2 cm) [4]. It is important to underscore that this distinction of AAC is not accepted by some authors: an analysis of the surveillance, epidemiology, and end results program of the national cancer institute of USA (SEER) database by Albores-Saavedra et al. [19] in 2009 demonstrated that AAC shows the same carcinogenic epidemiological pattern, independent of its histological diagnosis, suggesting a single population of AAC and similar or overlapping carcinogenic pathways. On the other hand, a matter of debate is the distinction of a third histological subtype of AAC, the mixed one (MIX) [17,20]. This third subset of AAC the shows intermediate histological and immunohistochemical features between INT and PB: thus, it is often difficult to definitively categorize MIX-AAC, and some authors preferred to classify tumours by the predominant type [3,11,21]. In fact, few studies focusing on MIX have been published. 

## 3. Prognosis of AACs

Since the definition of the different histological subtypes of AAC, several efforts in order to understand their prognostic impact have been done. Some studies [6,9,22,23,24,25,26,27] reported survival of patients with PB was poorer than those with INT. (Table 1), whereas others failed to find a significant difference between the two histological subtypes. Beghelli et al. [22], evaluating 89 resected AACs, had demonstrated that PB showed a higher proportion of poorly differentiated tumours (61%), whereas INT were well or moderately differentiated in 75% of cases (*p* < 0.006); median OS was 19 and 70 months for PB and INT, respectively (*p* = 0.027). Similar results were obtained by Carter et al. [9]: in multivariate analysis, the pancreatobiliary subtype, together with lymphovascular invasion, perineural invasion, and stage > III, was found to be a significant predictor of survival (HR 2.6; *p* = 0.01). A large multicentric retrospective study was published by Chang et al. [6], in which they assessed the prognostic value of histological subtypes in 208 patients from three independent cohorts of resected AACs: patients with PB showed a poor prognosis in all three cohorts (HR 3.34, 5.65, and 2.78; *p* < 0.05) in comparison with INT. Some studies also reported a relationship between histological subtype and risk of a disease’s recurrence. Kim et al. [24] showed that patients with PB had significantly poorer disease-free survival (DFS) than patients with INT (3- and 5-year DFS were 50.6% vs. 80.0% and 47.8% vs. 73.1%, respectively; *p* = 0.003). Similar results were obtained by a large multicentric retrospective study, in which median DFS was 25.3 months for PB vs. 58.9 months for INT (*p* < 0.05). 

On the other hand, other published studies failed to find a prognostic value of histological differentiation. Sessa et al. [28], in a retrospective evaluation of 53 resected AAC, demonstrated that no significant differences in terms of survival were found between PB and INT (HR 0.73; *p* = 0.08). Similar results were obtained by De Paiva et al. [29] and Bowitz et al. [30]: although INT showed better survival in the univariate analysis, histological subtype was not an independent predictor of poor prognosis. More importantly, a recent large multicentric retrospective trial evaluating 887 PD for AAC [31] found that histological differentiation was not an independent prognostic factor in multivariate analysis; N-stage (HR 3.30), perineural invasion (HR 1.50), and adjuvant chemotherapy (HR 0.69) were the only independent predictors of OS. Confirming these results, more recently, the same group developed and validated a nomogram model to predict prognosis after resection for AAC, including age, resection margin, tumour differentiation, and pathological T stage and N stage, excluding histological differentiation [32].

It is important to note that most contained only a small number of patients due to the rarity of the disease. A recent meta-analysis was published by Zhou Y et al. [33], with the aim to review the available literature and to investigate the prognostic role of histological subtypes of AAC. Twenty-three retrospective studies involving a total of 2234 patients were identified for inclusion, of whom 1021 (45.7%) had INT, and 899 (40.2%) had PB. Patients with PB had high rates of poor tumour differentiation (*p* < 0.001), lymph node metastasis (*p* < 0.001), vascular invasion (*p* < 0.001), perineural invasion (*p* < 0.001), and positive resection margins (*p* = 0.004), compared to patients with INT. Moreover, the authors demonstrated that PB predicted a worse OS (HR 1.84; *p* < 0.001) and DFS (HR 1.93; *p* = 0.004). The strength of this meta-analysis was that only articles reporting a multivariate-adjusted HR were pooled (mitigating the possibility of confounding variable influencing results) and that there was no significant heterogeneity or publication bias.

Even more difficult is the evaluation of the prognostic impact of mixed AAC, due to the few studies that have been published. Reid et al. [17] evaluated all three subtypes of AAC (PB, INTm and MIX): INTs were significantly largest (4.5 cm) compared with MIX (2.7 cm) and PB (2.2 cm) (*p* = 0.001); however, median OS survival was significantly different between INT and PB (80 vs. 41 months, respectively; *p* = 0.026) but not among PB and MIX (80 vs. 56 months, respectively; *p* = NS). The authors underlined the fact that the features of MIXs fell between INT and PB in all characteristics, but median OS (56 months) was closer to that of PB (40 months) than INT (80 months). Similar results were obtained by Zimmermann et al. [27] in a large patient cohort (n. 119): the mean OS of mixed AAC was intermediate to PB and INT (94.7 vs. 52.5 vs. 115, respectively; *p* < 0.001). 

We can conclude that it is extremely important to report the histological differentiation of AAC because the available literature seems to demonstrate that it has a significant clinical impact. If there is consensus that PB subtype has a more aggressive biological behaviour when compared with int, no conclusions on mix type could be reached, due to the few cases reported. Further multicentric studies on MIX are needed in order to evaluate its prognostic impact.

## 4. The Role of Adjuvant Therapy for Ampullary Cancer

The need of adjuvant therapy after resection for AAC represents probably the most debated aspect on this topic; this is due that there is not enough available evidence. Only one randomized controlled trial sponsored by the European Study Group for Pancreatic Cancer (ESPAC) has been published [34]. However, it is important to underscore that it was not designed specifically for ACC, but for all periampullary neoplasms (including pancreatic, distal common bile duct, and duodenal cancer). A subset analysis, evaluating the role of adjuvant treatment after resection for AAC, reported that 5-fluorouracil- or gemcitabine-based regimes did not significantly improve median OS (43.1 versus 35.2 months for treated and control group, respectively; *p* = 0.25). Several other studies on this topic have been published, but all of them were retrospective and took into account a low number of enrolled patients [35,36,37,38,39,40,41,42,43]. A meta-analysis and a systematic review with the aim to evaluate the role of adjuvant treatment for ACC have been published [44,45]. The meta-analysis, published in 2015, included 10 retrospective studies, for a total of 3361 evaluated patients: it demonstrated that adjuvant chemo-radiotherapy was associated with a lower risk of death (HR 0.75; *p* = 0.001) compared to surgery alone [44]. However, this meta-analysis also included randomized clinical trials on which AAC was grouped with other periampullary tumours (for example, the ESPAC-3 trial mentioned above) [34], making it difficult to ascertain the benefit of adjuvant therapy specifically in AAC. More recently, a systematic review by Bonet et al. [45] confirmed a benefit of adjuvant treatment for patients with locally advanced tumours, mainly for those with positive lymph nodes and T3-4 stages; in addition, the authors of some selected studies suggested that chemo-radiotherapy was more beneficial than chemotherapy alone. This systematic review, differently from meta-analysis mentioned above, included only studies focusing on AAC, even if all of them were retrospective. Due to this lack of evidence, even if adjuvant treatment seems to give promising results after resection for AAC, to date, there is no consensus, and, in fact, no international guidelines have been published until now.

The debate became even more complex if we took into account the distinction of AAC in the two histological subtypes (INT and PB). Theoretically, due to their different aetiology and biological behaviours, we could argue that the effect of adjuvant treatment should be evaluated differently for the two histological subtypes. However, the available literature is even more limited, and it is difficult to draw any conclusions. Recently, a multicentre cohort study by Bolm et al. [46], including 214 resected AACs, demonstrated that adjuvant therapy (mainly gemcitabine-based regimens) was associated with improved OS only in patients with PB and MIX, whereas no benefits were observed for INT. Similar results were obtained by Moekotte et al. [47] in a large international cohort study: they showed a significant survival benefit with adjuvant chemotherapy in PB and MIX patients but not for INT, using a propensity-score-matched analysis. All these studies had several biases, due to their retrospective natures: particularly, there was an extreme heterogeneity in terms of adopted chemotherapeutic regimens, and it is impossible to know which adjuvant treatment for each histological subtype should be adopted. Schiergens et al. [48] in a retrospective study demonstrated that patients with PB receiving adjuvant gemcitabine had improved OS (32 vs. 13 months, *p* = 0.013), whereas patients with INT had poorer survival with gemcitabine (35 vs. 112 months, *p* = 0.193). These findings could indicate that the response to different chemotherapies may have been dependent on histological subtypes of AAC: for PB, histologically more similar to pancreatic cancer, gemcitabine-based regimens could be indicated; conversely, for intestinal-type tumours, histologically similar to gastric or colorectal cancer, 5-fluororacil-based regimens could be more effective [34]. In conclusion, there is no consensus on the efficacy of adjuvant therapy, although some benefits could be reached when compared with the only-surgery group. Even more paucity of data are available on the role of adjuvant treatment according the different histological subtypes of AAC: it seems that adjuvant therapy (mainly gemcitabine-based regimens) could be effective particularly for PB and MIX. However, further prospective trials with a large number of patients are needed in order to confirm these data and to draw any conclusion on this topic.

## 5. The Role of Histological Biomarkers in AACs

**Figure 1:**
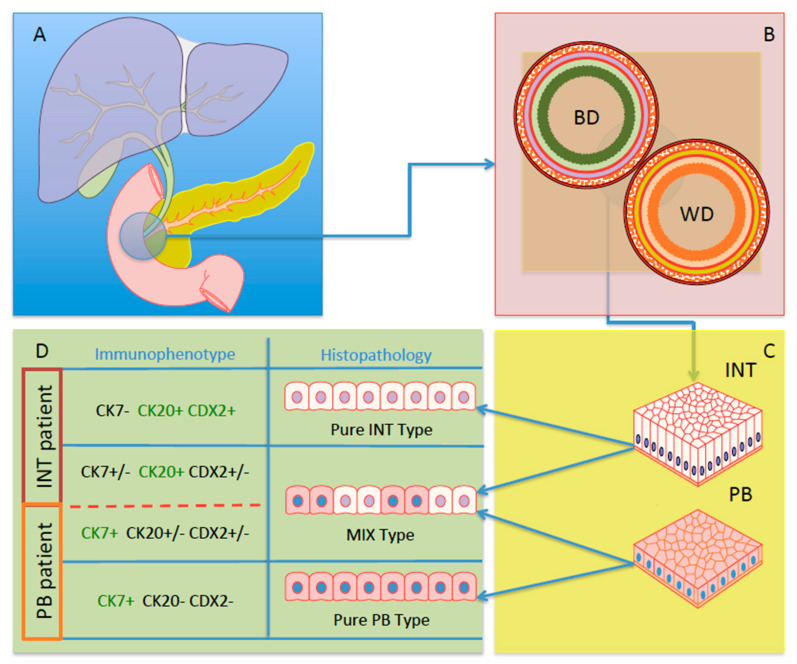
Schematic representation of HPB system and histo-molecular characteristic of AACs (**A**) Blue panel: HPB system. (**B**) Pink panel: Schematic structure of Bile duct (BD) and Wirsung Duct (WD) position inside the Vater’s ampulla region. (**C**) Yellow panel: Representation of Intestinal tissue (INT) and Pancreato-Biliary tissue (PB). (**D**) Green panel: Histo-molecular representation of INT, PB, and MIXED subtypes of AACs and binomial patients’ configurations.

Considering both clinical requirements and diagnostic purposes, some scientists were asked the following: “Can we improve ampullary carcinoma classification?”. As reported previously, some authors proposed in 2016 a sub-classification of AACs based on five subtypes [17]. On the other hand, the evidence of clinical management of these cancer patients have been suggesting a very useful, simple, and binomial classification, considering the predominant tissue genesis of AACs: INT or PB [10,11,21,49]. Nevertheless, it’s not easy to simplify this classification and the pathologists with the support of biomarkers, which are fundamental players in clinical management of ACC patients. Thus, Palmeri et al. demonstrated that analyses of three biomarkers (CK7, CK 20, and CDX-2) were a sufficient panel of useful molecules to classify all AACs into only two subgroups (INT and PB) and abrogate the presence of patients with subtype MIX [49]. In fact, the analyses performed by the scientists revealed that the global molecular expression of the three aforementioned markers were able to better stratify the patients, also impacting the OS curves of INT vs. PB [49]. In particular, the molecular characterization of these biomarkers was performed using an immunohistochemical approach, also indicating a feasible way not necessary in highly specialized centres. Furthermore, a few months ago, Luchini and Scarpa indicated immunohistochemistry (IHC) as the test of choice for the routine workflow in the clinical practice of PADC and AAC, in which IHC analyses acquire its diagnostic, prognostic, and predictive values [50] (Figure 1).

## 6. IHC and Its Diagnostic Role in AACs

In 1994, KIMURA et al. were the first to propose a sub-classification of AACs, based solely on histological findings [4]. In this study, the authors found a longer survival after surgery in patients with intestinal subtype compared to the pancreatobiliary subtype than in those with the pancreatobiliary type (PB) [51]. Ten years ago, some scientists verified the utility of IHC inside the histological classification of sub-types of AAC [7]. To understand the differences in terms of the histological classification, some authors proposed a different immuno-histochemical panel [6,12,17,19,22,27,28,31,52]. Tumours of the int group usually express cytokeratin-20 (CK20), caudal-type homeodomain transcription factor 2 (CDX2), and mucin-2 (MUC2) [33,53,54]. In contrast, the PB phenotype expresses ck7, muc1, and muc5a [9,26]. The hybrid cellular tumours show histomorphologic features of both subgroups [17,27,46,47]. Indeed, the immunohistochemical panels created to accurately define all cases remain a challenge for a binomial subclassification [8,30,55,56]. However, the triage of markers proposed through Palmeri et al.’s [49] analysis seem to be convincing of the presence of the “two side of the coin” only: INT and PB. Furthermore, rare evidences of metastatic lesion arising from AAC retained the immunostaining characterizations of CK7, CK20, and CDX2 [57,58,59,60]. Then, these three markers, featuring the histological examination, might constitute the routinary approach for the diagnostic statement of AACs.

## 7. IHC and Its Prognostic Role in AACs

AAC histological subtypes highlighted by IHC staining patterns have been shown to have prognostic significance. By the way, conflicting analyses concerning the distribution of the INT and PB subtypes of ampullary cancers have been reported. Survival of patients with PB tumours showing CK7/MUC1-positive staining and CDX2-negative staining showed a worse prognosis [6,8,61]. In a study including about 40 patients with ampullary adenocarcinoma who underwent PPPD surgery, half of them (18/37) had CK7-positive expressions inside their tumours. Multivariate analysis showed that CK7+/CK20− was a significant independent factor associated with poorer survival, whereas nodal positivity status was not predictive [61]. In another cohort, concerning 70 AAC patients whom underwent surgical resection, 25% of the patients with a PB subtype (MUC1/+ and CDX2/−) had significantly bad outcomes than those with an INT subtype (MUC1/− and CDX2/+), in which the median survival values were 16 vs. 116 months, respectively [6]. In an Italian report of 53 resected ampullary cancers, the expressions of CDX2 were found in 60% (32/53) of the total cases, where 30% of the PB type (9/30) and 100% INT type (23/23), showed a positive staining of the CDX2 protein. The expression of CDX2 was correlated with longer survival in patients, when the nuclear staining of tumour cells was over the 10% of malignant cells [28].

## 8. IHC and Its Predictive Role in AACs

Concerning the rarity and its frequency of ampullary adenocarcinoma, randomized clinical trials have claimed a way to establish a standard chemotherapy regimen for patient management. Looking at the cellular contents of lesions, both ampullary and duodenal adenocarcinomas have been treated in a similar way; the treatment regimens were close to cholangiocarcinoma or colon cancer. Pharmacological regimens included gemcitabine w/wo platinum compounds (cis- or oxali-) [62] or Capacitabine and Oxaliplatinum [63,64]. Another pharmacological treatment involved the fluoropyrimidine-based chemotherapy (FOLFOX or FOLFIRI). These therapies generally achieved a median survival of ≤1 year [63,64]. However, the choice of best supportive care is often not associated with a robust histological classification and or phenotypical landscape of AAC. Furthermore, some authors in 2019 changed the third line of chemotherapy in three AAC patients, looking for their immunophenotypical characterizations. A group of scientists performed analyses of CK7, CK20, MUC1, and CDX2 in tumour tissues of patients [62]. The IHC analyses were suggestive for the PB subtype for two patients and the INT subtype for the third one. So far, the clinicians decided to treat the PB patients using the nab-paclitaxel regimen and INT patients using FOLFOX chemotherapy. All three patients were considered non-responding subjects after two lines of chemotherapy treatments. The authors reported a prolonged survival for 2–3 years and a marked tumour reduction in respect to another study in which the patients were in the advanced stage of cancer. Nevertheless, the PB patient had the same immunophenotypical profile, (CK7/+ and CDX2/−), whereas the INT patient showed the following immuno-staining (CK7/+, CK20/+, and CDX2/+). In fact, in this study, the IHC analyses seemed to indicate to the clinicians not only the diagnosis of cancer, but the expression of markers seemed to drive the chemotherapy regimen in both the PB and INT patients [62]. 

## 9. Conclusions

AAC ambulatory tumours represent a group of heterogeneous malignancies, in which two subtypes are the major players: INT and PB. Interestingly, the source of this classification was not the histological study but the immunomarker characterization. However, their identification did not emerge immediately through histo-pathological analyses, where immunoimmune-phenotypic characterization seemed to be the goal of clinicians who managed to place patients on both sides of the river. Tissue biomarkers such as CK7, CK20, and CDX2 permitted establishing a prognostic and predictive forecast. However, CDX2 may be the “judge” of the fate of AAC patients. Indeed, CDX2 appears to be choosing between Dr. Jakill and Mr. Hide in AAC, but who is who? 

## 10. Future Perspectives

The choice-based evidence of an IHC panel selecting three specific markers (i.e., CDX-2, CK7, and CK20) seemed to support the pathologist for the binomial sub-classification of AAC. Indeed, the IHC evaluation represented a valid tool for diagnostic, prognostic, and predictive assessments for subtypes in AAC patients. Nevertheless, the recognition of only two subclasses of AAC, in a strict way, offered the homogenization of an infrequent cohort of patients, included often in a group of periampullary cancers. Furthermore, the creation of an automatic platform for biological marker revaluations opens the doors to the digital pathology discipline or a well we prefer to name “In silico Pathology”. This approach in the surgical pathology field could improve the decision-making step to providing an individualized treatment (according the INT or PB diagnostic decision), especially whether the patients should undergo primary surgery or neoadjuvant treatment.

This binomial molecular classification, according the immunophenotype (INT or PB), appears able to predict the clinical outcome in both INT and PB patients. The real future application of this technical triage, including the selection of bio-molecular markers, an automatized platform for analysis, and surgical procedure, may have found its major expression inside the preoperative biopsy of Vater’s ampulla. Here, AAC subtype classifications immediately split both INT and PB patients toward their tailored oncological treatments and a plan for the evaluation of the surgical approach.

## Figures and Tables

**Table 1:** Summary of principal clinical studies involving INT vs. PB subtypes.

Author	Ref.	N. of Patients	Histological Subtypes	Median OS	Median DFS
Beghelli et al.	[22]	89	Pb-AC: 56 (62.9%) In-AC: 28 (31.5%)	Pb-AC: 19 months In-AC: 70 months	-
Carter et al.	[9]	118	Pb-AC: 53 (44.9%) In-AC: 54 (45.8%)	Pb-AC: 22 months In-AC: 60 months	-
Ruemmele et al.	[23]	118	Pb-AC: 27 (22.9%) In-AC: 56 (47.5%)	Pb-AC: 41 months In-AC: 97 months	-
Kim et al.	[24]	104	Pb-AC: 62 (59.6%) In-AC: 42 (40.4%)	-	Pb-AC: 5y 47.8% In-AC: 5y 73.1%
Chang et al.	[6]	208	Pb-AC: 89 (42.8%) In-AC: 119 (57.2%)	Pb-AC: 22.0 months In-AC: 115.0 months	Pb-AC: 23.9 months In-AC: 69.9 months
Robert et al.	[25]	319	Pb-AC: 105 (32.9%) In-AC: 106 (32.2%)	-	Pb-AC: 25.3 months In-AC: 58.9 months
Schueneman et al.	[26]	163	Pb-AC: 75 (46.0%) In-AC: 50 (30.7%)	Pb-AC: 21.2 months In-AC: 106.4 months	-
Zimmermann et al.	[27]	170	Pb-AC: 69 (58%) In-AC: 41 (34.5%)	Pb-AC: 52.5 months In-AC: 115 months	-
